# Hesperetin Increases Lifespan and Antioxidant Ability Correlating with IIS, HSP, mtUPR, and JNK Pathways of Chronic Oxidative Stress in *Caenorhabditis elegans*

**DOI:** 10.3390/ijms252313148

**Published:** 2024-12-06

**Authors:** Run-Jia Wang, Ya-Jing Ni, Yan-Qiang Liu

**Affiliations:** College of Life Sciences, Nankai University, Tianjin 300071, China; wangrunjia202205@163.com (R.-J.W.); nyj2873065@126.com (Y.-J.N.)

**Keywords:** hesperetin, lifespan, antioxidative, transcriptomic metrics, *Caenorhabditis elegans*

## Abstract

Hesperetin (Hst) is a common citrus fruit flavonoid with antioxidant, anti-inflammatory, and anti-neurodegenerative effects. To explore the antioxidant and anti-aging effects and mechanisms of Hst, we induced chronic oxidative stress in *Caenorhabditis elegans (C. elegans)* using low-concentration H_2_O_2_ and examined its effects on lifespan, healthy life index, reactive oxygen species (ROS), antioxidant enzymes, and transcriptomic metrics. Hst significantly prolonged lifespan, increased body bending and pharyngeal pumping frequency, decreased ROS accumulation, and increased antioxidant enzyme activity in normal and stressed *C. elegans*. Hst significantly upregulated *daf-18*, *daf-16*, *gst-2*, *gst-3*, *gst-4*, *gst-39*, *hsp-16.11*, *sip-1*, *clpp-1*, and *dve-1* and downregulated *ist-1* and *kgb-1* mRNAs in stressed *C. elegans*. These genes are involved in the insulin/insulin-like growth factor-1 signaling (IIS), heat shock protein (HSP), mitochondrial unfolded protein response (mtUPR), and c-Jun N-terminal kinase (JNK) pathways. In summary, Hst increases lifespan and antioxidant ability, correlating with these pathways, during chronic oxidative stress in *C. elegans*.

## 1. Introduction

Aging is a degenerative process that eventually leads to functional damage or even the death of cells, tissues, and whole organisms [1]. This process is accompanied by chronic disorders in humans including neurodegenerative, cardiovascular, metabolic, musculoskeletal, and immune system diseases [2]. In recent years, with the increasingly aging population and high incidence of age-related diseases, delaying the aging process, extending a healthy lifespan, and scientifically managing aging have become important issues.

A variety of hypotheses have been proposed regarding senescence development, among which the free radical hypothesis is a major theory [3,4]. Reactive oxygen species (ROS) are the most common free radicals found in living organisms. Under normal metabolic conditions, ROS production activates the expression of the nuclear factor erythroid-derived 2-like 2 (Nrf2) signaling pathways and antioxidant enzymes, such as catalase (CAT), superoxide dismutase (SOD), and glutathione S-transferase (GST), which create a dynamic balance of the ROS content in the body [5]. However, with increasing age, the activity of antioxidant enzymes in the body decreases, and excess ROS accumulate. Excessive ROS induce oxidative stress, disrupt intracellular REDOX homeostasis, and cause peroxidative damage to lipids, proteins, and DNA, thus leading to age-related diseases such as neurodegenerative diseases, cardiovascular diseases, cancer, and even death [6]. Therefore, antioxidants are particularly important due to their anti-aging effects.

**Figure 1:**
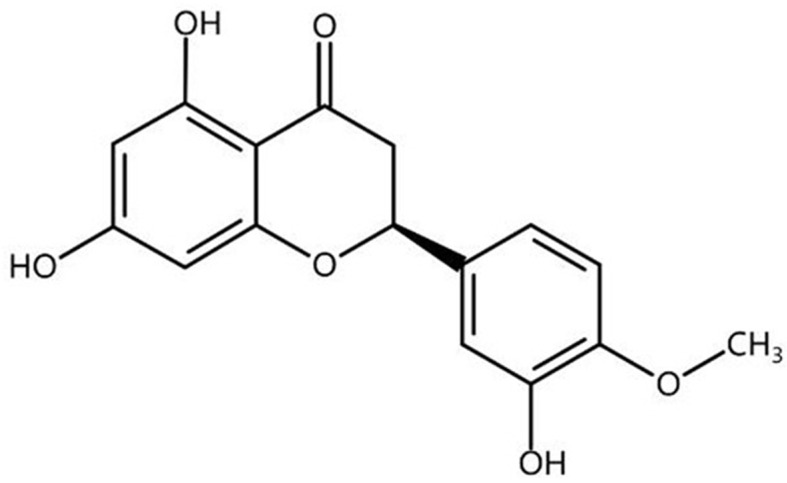
The chemical skeletal structural formula of hesperetin. Chemical formula: C_16_H_14_O_6_; molecular weight: 304.2713.

Enzymatic and non-enzymatic antioxidants can prevent free radicals from damaging biological macromolecules such as proteins, nucleic acids, and lipids. Endogenous enzymatic antioxidants include SOD, CAT, and GST [7]. Non-enzymatic antioxidants are mostly exogenous natural compounds with antioxidant properties and free radical-scavenging effects, among which flavonoids are widely used [8]. Hesperetin (Hst, the chemical skeletal structural formula [9] is shown in Figure 1) is one of the most common flavonoids found in citrus fruits such as oranges, tangerines, grapefruits, lemons, and limes, and is the aglycone form of hesperidin. Hst has antioxidant [10,11,12,13], anti-inflammatory [10,11,12], antiviral [14], and other physiological and pharmacological functions. Hst reduces acute lipopolysaccharide-induced kidney injury by increasing the CAT, GST, and SOD levels in mice [10]. It also inhibits the expression of genes related to nuclear factor kappa-B (NF-κB) signaling to reduce inflammation in activated BV-2 microglia [12]. Hst also has antidiabetic [9,15], anticancer [16,17,18], antineurodegenerative, and neuroprotective functions [19,20,21,22]. Hst significantly decreases inflammatory factors such as tumor necrosis factor-α (TNF-α) and interleukin 6 (IL-6) in high-glucose cell models [15]. Hst also inhibits mammary ball formation and migration in breast cancer cells by increasing the mRNA levels of *p53*, *NOTCH1*, and peroxisome proliferator activated receptor gamma (*PPARG*) [17]. In addition, Hst improves cognitive and memory decline in Aβ_1–42_ induced AD mice [20].

*Caenorhabditis elegans* was first established as a model organism by Sydney Brenner in the 1960s [23] and is now widely used in the fields of life sciences. *C. elegans* has been used in aging and anti-aging research because of its short lifespan and high fertility. *C. elegans* has nearly 20,000 protein-coding genes, about 60–80% of which are homologous to human genes including those involved in aging, apoptosis, metabolism, signal transduction, and the cell cycle [24]. A variety of signaling pathways associated with aging including insulin/insulin-like growth factor signaling-1 (IIS), mitogen-activated protein kinase (MAPK), mechanistic target of rapamycin (mTOR), mitochondrial unfolded protein response (mtUPR), heat shock protein (HSP), and c-Jun N-terminal kinase (JNK) pathways have been identified in *C. elegans*. In eukaryotes, the IIS pathway is involved in growth, metabolism, aging, and stress response. Inhibition of the IIS pathway activates the transcription factor DAF-16/FOXO and then activates the expression of downstream target genes, such as antioxidant enzymes and other stress- and life-related genes, thereby prolonging lifespan and enhancing the anti-stress ability [25]. MAPK regulate exogenous oxidative stress and inflammatory factors [26]. Under stress conditions, the MAPK pathway is activated, and the transcription factor SKN-1/Nrf2 is phosphorylated to promote the expression of detoxification enzyme genes [27].

Oxidative stress is a key factor leading to aging. Although a few studies have found that Hst has antioxidant and anti-aging effects, the specific molecular mechanism of the effects of Hst is still unclear and needs to be thoroughly studied. The present study aimed to induce chronic oxidative stress *C. elegans*, observe the effects of Hst on aging, ROS levels, antioxidant enzyme activity, and transcriptomic metrics of oxidative stress *C. elegans*, and understand the anti-aging effect and its possible mechanism in oxidative stress status. Our study hopes to provide a novel explanation on the underlying molecular mechanism of the Hst anti-aging effect and support its clinical application for controlling age-related diseases.

## 2. Results

### 2.1. Optimal Hst Treatment Concentration Based on Its Effect on Healthy Lifespan Indices and Oxidative Stress Indicators in Normal C. elegans

**Figure 2:**
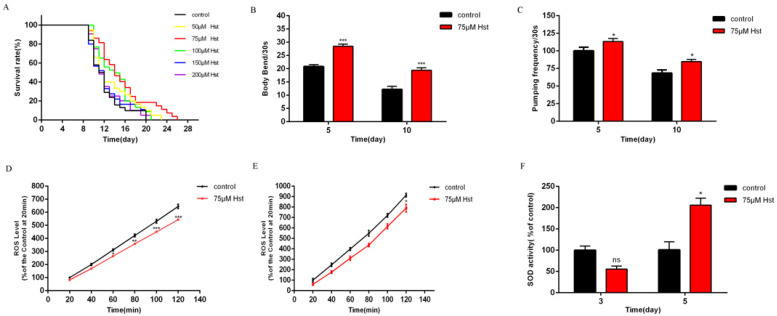
The optimal Hst treatment concentration based on lifespan and its effect on healthy lifespan indices and indicators of oxidative stress in normal *C. elegans.* (**A**) Different concentration of Hst caused lifespan changes of normal *C. elegans.* (**B**) The frequency of body bending in 30 s of *C. elegans* treated with 0 μM and 75 μM Hst for 5 d and 10 d. (**C**) The frequency of pharyngeal pumping in 30 s of *C. elegans* treated with 0 μM and 75 μM Hst for 5 d and 10 d. (**D**) ROS fluorescence dynamics of *C. elegans* treated with 0 μM and 75 μM Hst for 3 d. (**E**) ROS fluorescence dynamics of *C. elegans* treated with 0 μM and 75 μM Hst for 5 d. (**F**) SOD activity of *C. elegans* treated with 0 μM and 75 μM Hst for 3 d and 5 d. Data were derived from three independent replicates and presented in the mean ± SEM, * *p* < 0.05, ** *p* < 0.01, *** *p* < 0.001 and ^ns^ *p* > 0.05 compared with the control (0 μM Hst).

Figure 2 shows the optimal Hst concentration based on lifespan, and its effect on healthy lifespan indices and oxidative stress indicators in normal *C. elegans*. First, normal *C. elegans* were respectively treated with 0 μM (control), 50 μM, 75 μM, 100 μM, 150 μM, and 200 μM Hst, and different lifespan curves were observed, as shown in Figure 2A. Compared with that of the control group, the average and maximum lifespans of *C. elegans* treated with 75 μM Hst were extended by 16.28% (*p* < 0.05) and 27.27% (*p* < 0.01), respectively. The mean and maximum lifespans of the other Hst-treated nematodes were extended, but the differences were not significant. Therefore, 75 μM Hst was recognized as being the optimal treatment concentration based on the lifespan in normal *C. elegans*.

Then, the effect of 75 μM Hst on the healthy lifespan indices and indicators of oxidative stress in normal *C. elegans* was determined. Normal *C. elegans* were respectively treated with 0 μM (control) and 75 μM Hst, and their different healthy lifespan indices are shown in Figure 2B,C. Compared with the control, 75 μM Hst treatment for 5 and 10 d increased the frequency of body bending by 36.82% (*p* < 0.001) and 59.57% (*p* < 0.001), respectively; additionally, it increased the frequency of pharyngeal pumping by 13.06% (*p* < 0.05) and 23.72% (*p* < 0.05), respectively. The ROS and SOD contents in the control and 75 μM Hst-treated *C. elegans* are shown in Figure 2D–F. Compared with the control, *C. elegans* treated with 75 μM Hst for 3 d and 5 d showed 15.18% (t = 60 min, *p* < 0.05) and 13.53% (t = 120 min, *p* < 0.05) lower ROS levels, respectively, whereas 75 μM Hst treatment for 5 d increased the SOD activity by 104.67% (*p* < 0.05). These results suggest that 75 μM Hst may increase the lifespan and antioxidative ability of normal *C. elegans* by decreasing the ROS levels and increasing the SOD activity in vivo.

Based on the results of the above experiments, 75 μM was chosen as the Hst treatment concentration for follow-up experiments.

### 2.2. Hst Prolonged the Lifespan of C. elegans Under Chronic Oxidative Stress

**Figure 3:**
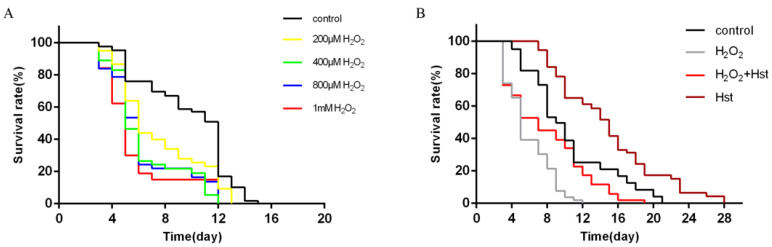
Effects of H_2_O_2_ on the lifespan of *C. elegans* and Hst on the lifespan of *C. elegans* under chronic oxidative stress. (**A**) Different *C. elegans* lifespan resulted from different doses of H_2_O_2_ treatment. (**B**) Effect of Hst on the lifespan of *C. elegans* under chronic oxidative stress. Data were derived from three independent replicates.

To determine the H_2_O_2_ concentration to establish the chronic oxidative stress *C. elegans* model, we treated the animals with 0 μM (control), 200 μM, 400 μM, 800 μM, and 1 mM H_2_O_2_ to obtain various lifespan curves (Figure 3A). Compared to the control, 200 μM, 400 μM, 800 μM, and 1 mM H_2_O_2_ decreased the average lifespan by 12.64%, 21.61% (*p* < 0.05), 27.86% (*p* < 0.01), and 37.11% (*p* < 0.001), respectively. In addition, the maximum lifespan of the 1 mM H_2_O_2_-treated nematodes was 12 d, which is consistent with the expected chronic oxidative stress treatment required to simulate older nematodes. Therefore, 1 mM H_2_O_2_ was considered to be the optimal concentration for chronic oxidative stress treatment.

The effect of Hst on the lifespan of *C. elegans* under chronic oxidative stress was observed. *C. elegans* were treated with 0 μM (control), 1 mM H_2_O_2_, 1 mM H_2_O_2_ + 75 μM Hst, and only 75 μM Hst simultaneously, and the different lifespan curves are shown in Figure 3B. Compared to the control, 1 mM H_2_O_2_ treatment decreased the average and maximum lifespan of *C. elegans* by 37.73% (*p* < 0.01) and 40% (*p* < 0.001), respectively, whereas simultaneous 1 mM H_2_O_2_ and 75 μM Hst treatment showed no significant difference. Compared with the 1 mM H_2_O_2_ treatment, simultaneous 1 mM H_2_O_2_ and 75 μM Hst increased the average and maximum lifespan of *C. elegans* by 43.94% (*p* < 0.01) and 33.3% (*p* < 0.01), respectively, whereas only 75 μM Hst resulted in the longest average and maximum lifespan of C. elegans. Therefore, 75 μM Hst prolonged the lifespan of normal and chronic oxidative stress *C. elegans*.

### 2.3. Hst Increased the Healthy Lifespan Indices in C. elegans Under Chronic Oxidative Stress

**Figure 4:**
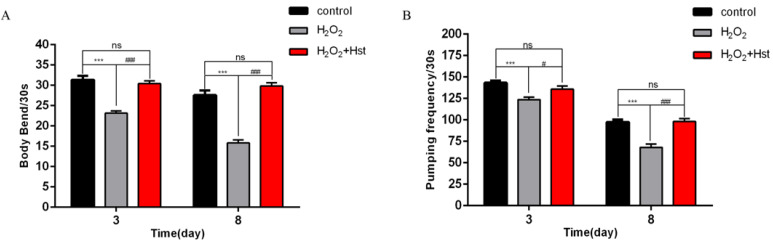
Effects of 75 μM Hst on the healthy lifespan indices of *C. elegans* under chronic oxidative stress. (**A**) The frequency of the body bending of *C. elegans* in the control, H_2_O_2_, and H_2_O_2_ + Hst treatment. (**B**) The frequency of the pharyngeal pumping of *C. elegans* in the control, H_2_O_2_, and H_2_O_2_ + Hst treatment. Data were derived from three independent replicates and presented in mean ± SEM, *** *p* < 0.001 and ^ns^ *p* > 0.05 compared with the control (0 μM Hst); ^#^ *p* < 0.05 and ^###^ *p* < 0.001 compared with the H_2_O_2_ treatment.

*C. elegans* were respectively treated with 0 μM (control), 1 mM H_2_O_2_, and 1 mM H_2_O_2_ + 75 μM Hst (simultaneously), and their different healthy lifespan indices are shown in Figure 4. Compared with the control, treating with 1 mM H_2_O_2_ for 3 d and 8 d decreased the frequency of the body bending of *C. elegans* by 26.06% (*p* < 0.001) and 42.60% (*p* < 0.001), respectively, and decreased the frequency of pharyngeal pumping by 13.78% (*p* < 0.001) and 30.22% (*p* < 0.001), respectively. Simultaneous treatment with 1 mM H_2_O_2_ and 75 μM Hst showed no significant difference (*p* > 0.05) in terms of the frequency of body bending and pharyngeal pumping of *C. elegans*. Compared with the 1 mM H_2_O_2_ treatment, simultaneous treatment with 1 mM H_2_O_2_ and 75 μM Hst for 3 d and 8 d increased the frequency of the body bending of *C. elegans* by 31.29% (*p* < 0.001) and 88.42% (*p* < 0.001), respectively; additionally, the frequency of pharyngeal pumping increased by 10.00% (*p* < 0.05) and 44.29% (*p* < 0.001), respectively. The results showed that 75 μM Hst increased the healthy lifespan indices of *C. elegans* under chronic oxidative stress.

### 2.4. Hst Increased SOD and CAT Activity and Decreased the ROS Accumulation in a C. elegans Model of Chronic Oxidative Stress

**Figure 5:**
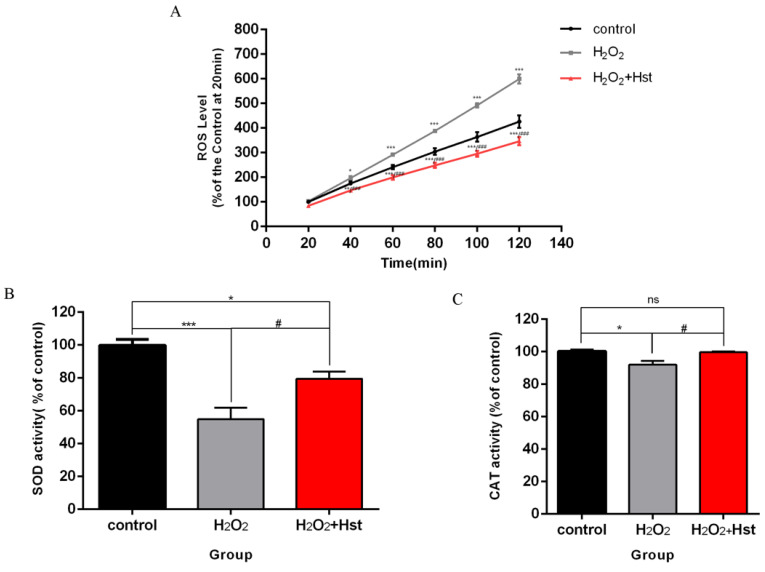
Effects of 75 μM Hst on ROS, SOD, and CAT in chronic oxidative stress *C. elegans*. (**A**) ROS fluorescence dynamics of *C. elegans* in different treatments. (**B**) SOD activity of *C. elegans* in different treatments. (**C**) CAT activity of *C. elegans* in different treatments. Data were derived from three independent replicates and presented in mean ± SEM, * *p* < 0.05, ** *p* < 0.01, *** *p* < 0.001 and ^ns^ *p* > 0.05 compared with the control (0 μM Hst); ^#^ *p* < 0.05 and ^###^ *p* < 0.001 compared with the H_2_O_2_ treatments.

*C. elegans* were respectively treated with 0 μM (control), 1 mM H_2_O_2_, and 1 mM H_2_O_2_ + 75 μM Hst (simultaneously), and their ROS, SOD, and CAT contents are shown in Figure 5. Compared with the control, a 3 d treatment with 1 mM H_2_O_2_ increased the ROS level of *C. elegans* by 12.23% (at 40 min, *p* < 0.05), and simultaneous treatment with 1 mM H_2_O_2_ and 75 μM Hst decreased the ROS level of *C. elegans* by 16.15% (at 40 min, *p* < 0.01). Compared with 1 mM H_2_O_2_ treatment, simultaneous treatment with 1 mM H_2_O_2_ and 75 μM Hst decreased the ROS level of *C. elegans* by 25.29% (at 40 min, *p* < 0.001).

Compared with the control, treatment with 1 mM H_2_O_2_ for 3 d decreased the activity of the SOD and CAT of *C. elegans* by 44.93% (*p* < 0.001) and 8.13% (*p* < 0.05), respectively, and simultaneous treatment with 1 mM H_2_O_2_ and 75 μM Hst decreased the activity of SOD of *C. elegans* by 20.37% (*p* < 0.05). Compared with 1 mM H_2_O_2_ treatment, simultaneous treatment with 1 mM H_2_O_2_ and 75 μM Hst increased the SOD and CAT activity levels of *C. elegans* by 24.57% (*p* < 0.05) and 7.57% (*p* < 0.05), respectively.

These results indicate that 75 μM Hst decreased the ROS levels and increased the SOD and CAT activity in chronic oxidative stress *C. elegans*.

### 2.5. Hst Changed the Expression of Aging and Metabolism-Related Transcripts in C. elegans Under Chronic Oxidative Stress

**Figure 6:**
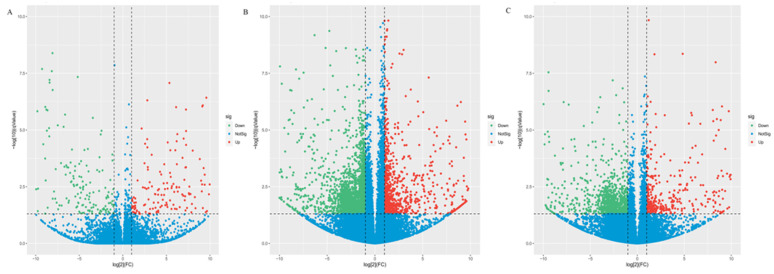
Volcano map of the differentially expressed transcripts. (**A**) Differentially expressed transcripts between the control and H_2_O_2_ treatment. (**B**) Differentially expressed transcripts between the control and H_2_O_2_ + Hst treatment. (**C**) Differentially expressed transcripts between the H_2_O_2_ and H_2_O_2_ + Hst treatment. Each point represents a gene, and the horizontal coordinate represents the logarithmic value log_2_ (fold change) of the expression difference of genes between the two groups. The ordinate represents the *p*adj (*q*) value of the change in gene expression minus the value log_10_ (*p*adj). The greater the absolute value of the horizontal coordinate, the greater the difference of the expression multiple between the two groups. The larger the ordinate value, the more significant the differential expression. Downregulated differential genes are shown with green dots, upregulated differential genes are shown with red dots, and unchanged genes are shown with blue dots. UP/DOWN is the number of differentially expressed transcripts upregulated or downregulated between two treatments.

The differentially expressed transcripts are shown in Figure 6 and Table 1. Compared with the control, H_2_O_2_ treatment resulted in 574 differentially expressed transcripts including 273 significantly upregulated and 301 significantly downregulated transcripts in *C. elegans*; meanwhile, the H_2_O_2_ + Hst treatment resulted in 3590 differentially expressed transcripts including 2545 significantly upregulated and 1045 significantly downregulated transcripts in *C. elegans*. Compared with the H_2_O_2_ treatment, the H_2_O_2_ + Hst treatment resulted in 1786 differentially expressed transcripts including 1265 significant upregulated and 521 significantly downregulated transcripts in *C. elegans*.

Differentially expressed transcripts were analyzed using the NCBI and Worm databases, some of which are shown in Table 2. We found that *daf-16* encoding the forkhead box O1 (FOXO1) homolog *DAF-16* [28,29], *gst-4* encoding GST, *hsp-16.11* encoding heat stress protein, *let-363* encoding the kinase homolog of rapamycin, *clpp-1* encoding the homolog of caseinolytic mitochondrial matrix peptidase proteolytic subunit, *pmk-2* encoding the homolog of p38 mitogen-activated protein kinase, among others, were present, which are related to the IIS, HSP, mtUPR, mTOR, and MAPK pathways. These results indicate that Hst regulates aging and metabolic processes of *C. elegans* under chronic oxidative stress.

### 2.6. Hst Activated the Longevity Regulating Pathway of C. elegans Under Chronic Oxidative Stress

**Figure 7:**
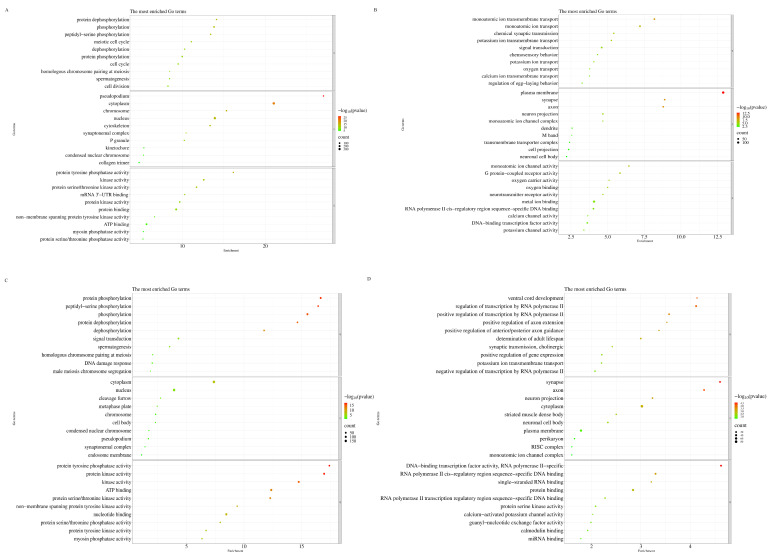
GO analysis of differentially expressed transcripts in the H_2_O_2_ + Hst treatment compared with the control and H_2_O_2_ treatment, respectively (**A**) GO analysis of upregulated differentially expressed transcripts in H_2_O_2_ + Hst treatment compared with the control. (**B**) GO analysis of down-regulated differentially expressed transcripts in the H_2_O_2_+Hst treatment compared with the control. (**C**) GO analysis of upregulated differentially expressed transcripts in the H_2_O_2_ + Hst treatment compared with the H_2_O_2_ treatment. (**D**) GO analysis of downregulated differentially expressed transcripts in the H_2_O_2_ + Hst treatment compared with the H_2_O_2_ treatment. This figure selected the top 10 GO terms to plot according to the enrichment order, and displayed all if less than 10. The vertical coordinate represents the GO term, and the horizontal coordinate represents the *p*-value of each term minus the value −log_10_ (*p*). The circle represented represents the transcripts enriched in each term, and the size of the circle represents the number of transcripts. The circle color indicates the significance of the term, and the redder the color, the more significant the term.

GO analysis was performed on the differentially expressed transcripts of the H_2_O_2_ + Hst treatment group compared to those of the control and H_2_O_2_ treatment groups. As shown in Figure 7, the upregulated transcripts following H_2_O_2_ + Hst treatment were significantly enriched for protein phosphorylation and dephosphorylation, peptidyl-serine phosphorylation, protein tyrosine phosphatase activity, protein kinase activity, and protein serine/threonine kinase activity terms, which were enriched in the cytoplasm cellular component. The downregulated transcripts of the H_2_O_2_ + Hst treatment were significantly enriched for the monoatomic ion transmembrane transport, chemical synaptic transmission, monoatomic ion channel activity, G protein-coupled receptors, regulation of transcription by RNA polymerase II, DNA-binding transcription factor activity, and RNA polymerase II-specificity terms; these were also enriched in the synapse cellular component.

**Figure 8:**
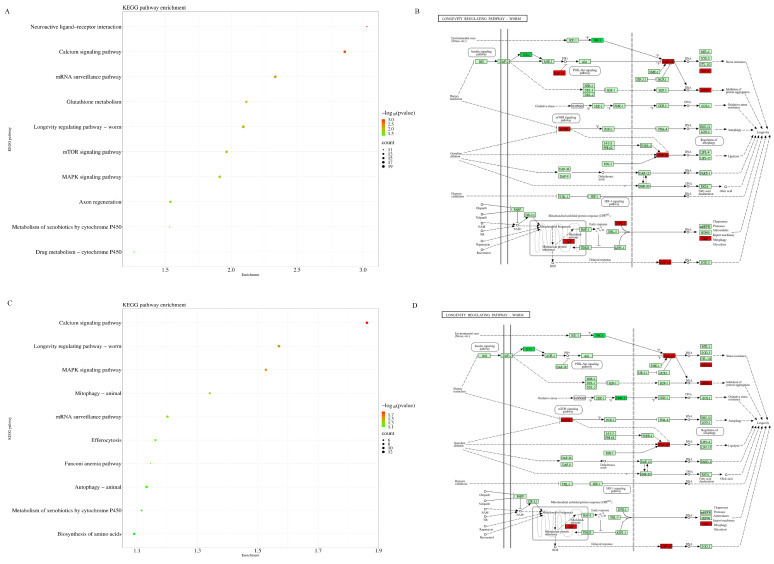
KEGG analysis of differentially expressed transcripts in the H_2_O_2_ + Hst treatment compared with the control and H_2_O_2_ treatment, respectively (**A**) KEGG analysis of differentially expressed transcripts in the H_2_O_2_ + Hst treatment compared with the control. (**B**) The differential transcripts of the H_2_O_2_ + Hst treatment compared with the control were enriched in the longevity regulating pathway of nematodes, where red shows those upregulated and dark green are those downregulated. (**C**) KEGG analysis of differentially expressed transcripts in the H_2_O_2_ + Hst treatment compared with the H_2_O_2_ treatment. (**D**) The differential transcripts of the H_2_O_2_ + Hst treatment compared with the H_2_O_2_ treatment were enriched in the longevity regulating pathway of nematodes.

KEGG analysis was performed on the differentially expressed transcripts of the H_2_O_2_ + Hst treatment compared with the control and H_2_O_2_ treatment, respectively. As shown in Figure 8, the differentially expressed transcripts in the H_2_O_2_ + Hst treatment group were significantly enriched in the calcium signaling, longevity regulating pathway-worm, and MAPK signaling pathways.

The longevity regulating pathway (KEGG-ID: ko04212) of the H_2_O_2_ + Hst treatment is shown in Figure 8. We found that the differentially expressed transcripts were enriched in the IIS, HSP, mtUPR, JNK, MAPK, and mTOR pathways, and their expressions are shown in Table 3. In the IIS pathway, the expression of *ist-1* encoding the insulin receptor substrate was downregulated, the expression of *daf-18* encoding phosphatidylinositol 3,4,5-trisphosphate 3-phosphatase and dual-specificity protein phosphatase was upregulated, and *daf-16*, *gst-2*, *gst-3*, *gst-4*, *gst-8*, and *gst-39* were upregulated. In the HSP pathway, the expression of *sip-1* and *hsp-16.11*, encoding heat stress proteins, was upregulated. In the mtUPR pathway, the expression levels of *clpp-1* and *dve-1*, encoding the caseinolytic mitochondrial matrix peptidase proteolytic subunit (ClpP-1) and a protein-folding chaperone, respectively, were upregulated. In the JNK pathway, the expression of *kgb-1*, homologous to JNK-1, which encodes c-Jun N-terminal kinase in *C. elegans*, was downregulated. In the MAPK pathway, the expression of *pmk-2* was downregulated. In the mTOR pathway, *let-363*, which is homologous to mTOR, was upregulated. In addition, the expression of *daf-12*, which encodes the nuclear hormone receptor, was downregulated.

## 3. Discussion

In this study, we found that the effect of Hst on the survival rate of *C. elegans* was greater at 75 μM than at 50 μM and 100 μM, indicating that the effect of Hst on the longevity of *C. elegans* increased in a dose-dependent way between 50 μM and 75 μM. The effect of Hst at 100 μM was higher than that at 50 μM, which might indicate that Hst at a low concentration had less effect on the lifespan extension of *C. elegans*. Therefore, we first determined that 75 μM Hst was the optimal treatment concentration by observing the lifespan, the frequency of body bending and pharyngeal pumping, and the ROS and SOD levels in normal *C. elegans*. Then, we found that 75 μM Hst significantly prolonged the lifespan, increased the frequency of body bending and pharyngeal pumping, decreased ROS accumulation, and increased antioxidant enzyme activity in *C. elegans* under chronic oxidative stress. RNA-Seq showed that 75 μM Hst significantly upregulated the mRNA transcript levels of *daf-18*, *daf-16*, *gst-2*, *gst-3*, *gst-4*, *gst-39*, *hsp-16.11*, *sip-1*, *clpp-1*, and *dve-1* and significantly downregulated those of *ist-1* and *kgb-1* in H_2_O_2_-induced *C. elegans*. These genes are involved in the IIS, HSP, mtUPR, and JNK pathways. These results suggest that Hst increases the lifespan and antioxidative ability, correlating with its regulation of the IIS, HSP, mtUPR, and JNK pathways during chronic oxidative stress in *C. elegans*.

Previous studies have shown that Hst can prolong the lifespan and delay the aging process in naturally aging mice [30]. This study also demonstrates that 75 μM Hst significantly prolongs the lifespan of *C. elegans* under normal and chronic oxidative stress, indicating that Hst exhibits similar anti-aging effects for *C. elegans* as those seen in mice.

Aging is frequently accompanied by impaired muscle integrity and functionality, resulting in the deterioration of motor ability [31]. This, together with swallowing ability, constitutes one of the criteria for assessing a healthy lifespan [32]. The present experimental results showed that 75 μM Hst significantly increased the frequency of body bending and pharyngeal pumping of chronic oxidative stress *C. elegans*, suggesting that Hst increases a healthy lifespan and delays the aging process of *C. elegans* under chronic oxidative stress.

Under oxidative stress conditions, endogenous or exogenous ROS can damage the proteins, lipids, and nucleic acid components of cells. Antioxidant enzymes can restore cellular homeostasis by removing excess ROS [33]. Flavonoids have antioxidant and free radical scavenging effects [34]. Naringin increases the enzymatic activities of SOD, CAT, and GST, thereby improving the oxidative stress resistance of *C. elegans* [35]. Myricetin modulates oxidative stress by increasing the radical scavenging ability in *C. elegans* [36]. This study found that 75 μM Hst decreased ROS levels and increased *C. elegans* antioxidant activity under chronic oxidative stress, suggesting that Hst can improve the antioxidative ability of *C. elegans* under such conditions.

Several pathways related to longevity regulating have been identified in *C. elegans*. The IIS pathway was the first that was found to be involved in regulating the aging process in animals, and is activated by insulin binding to the membrane receptor tyrosine kinase [37]. *C. elegans* has only one homologous insulin-like growth factor 1 (IGF-1) receptor, DAF-2. DAF-2 phosphorylates DAF-16 through a cascade reaction inhibiting its entry into the nucleus [29]. If *daf-2* expression is inhibited, the nuclear translocation of DAF-16 can be promoted, and the expression of stress-response-related genes can be activated to delay aging. In vertebrates and fruit flies, adaptor proteins in the IIS pathway connect growth factor receptors to intracellular signaling pathways. Ligands bind to and activate the tyrosine kinase activity of insulin and IGF-I receptors, resulting in the insulin receptor substrate (IRS) becoming phosphorylated. Phosphotyrosine on IRS is the binding site of downstream targets such as MAPK [38]. *Ist-1* encodes the insulin receptor substrate protein IST-1 in *C. elegans*, which binds to DAF-2 to phosphorylate DAF-16. Previous studies have speculated that DAF-2 activates IST-1 to transmit signals [39,40]. In this study, *ist-1* was downregulated and *daf-16* was upregulated in *C. elegans* simultaneously treated with H_2_O_2_ and Hst, suggesting that Hst may inhibit the expression of *ist-1* and impede the phosphorylation of DAF-16, thereby prolonging the lifespan of *C. elegans* under chronic oxidative stress.

*Daf-18* encodes the human tumor suppressor PTEN homolog, DAF-18, which exhibits 3-phosphatase activity against phosphatidylinositol 3,4,5-trisphosphate (PIP3). Studies have shown that DAF-18 plays a role in limiting Akt activation by decreasing the level of PIP3 produced by AGE-1, thereby regulating *daf-16* transcription [41]. DAF-18 acts in a tissue-specific manner to regulate lifespan, which depends on the activation of DAF-16 [42]. In the present study, the expression of *daf-18* and *daf-16* was upregulated in *C. elegans* simultaneously treated with H_2_O_2_ and Hst, suggesting that Hst promotes the expression of *daf-18* and impedes the phosphorylation of DAF-16, thereby prolonging the lifespan of *C. elegans* under chronic oxidative stress.

The transcription factor DAF-16 is a negative target of the IIS cascade, which is widely involved in the regulation of physiological processes including longevity, stress, metabolism, and protein homeostasis. DAF-16 is a potential target for various anti-aging substances used to treat human aging and aging-related diseases [29,37]. DAF-16 can activate the expression of genes encoding antioxidant enzymes, such as *sod-3* encoding SOD, *clt-1* and *clt-2* encoding CAT, and *gst-4* encoding GST, thereby improving the antioxidant ability and prolonging the lifespan of *C. elegans*. In the present study, *gst-4*, *gst-2*, *gst-3*, *gst-8*, and *gst-39* were upregulated in *C. elegans* simultaneously treated with H_2_O_2_ and Hst. These results suggest that Hst improves the antioxidant ability and prolongs lifespan via the IIS pathway during chronic oxidative stress in *C. elegans*.

As molecular chaperones, HSPs protect proteins from cellular stress and prevent protein aggregation and decomposition, which are crucial for organisms to adapt to environmental changes and maintain homeostasis [43]. Studies have shown that HSPs are the key factors in improving stress resistance and prolonging the lifespan of *C. elegans*. Carnosic acid improves stress resistance and prolongs the lifespan of *C. elegans* by upregulating the expression of *hsp-16.1* and *hsp-16.2* [44]; likewise, 6-gingerol prolongs lifespan and enhances stress resistance in *C. elegans* via *hsp-16.2* [45]. In the present study, the expression of *hsp-16.11* and *sip-1* was upregulated in *C. elegans* simultaneously treated with H_2_O_2_ and Hst, suggesting that Hst may prolong the lifespan of *C. elegans* under chronic oxidative stress by regulating the HSP pathway.

The roles of the mitochondria in energy production, calcium homeostasis, apoptosis, and ROS balance are essential for cell survival [46]. Senescent cells often accumulate large amounts of unfolded or misfolded proteins in the mitochondria. Therefore, mitochondrial dysfunction is a hallmark of aging [47]. MtUPR regulates mitochondrial protein homeostasis. Under mitochondrial stress conditions, ClpP-1, encoded by *clpp-1*, can be activated to degrade unfolded or misfolded proteins in the mitochondria into short peptides, and the transcription factor defective proventriculus 1 (DVE-1), encoded by *dve-1*, can be translocated to the nucleus to activate mtUPR-related gene expression [48,49,50]. Studies have linked mtUPR to longevity; activated mtUPR specifically prolongs lifespan and enhances the anti-stress ability of *C. elegans* [51]. NAD(+) prolongs the lifespan of *C. elegans* by activating mtUPR [52]. In this study, the expression of *clpp-1* and *dve-1* was significantly upregulated by Hst during chronic oxidative stress in *C. elegans*, suggesting that Hst may prolong their lifespan by activating mtUPR to remove unfolded or misfolded proteins.

The JNK pathway is a key pathway that protects cells from stress damage and prolongs their lifespan [53]. In *C. elegans*, the JNK pathway is thought to exert an antagonistic effect on the IIS pathway during the regulation of DAF-16. JNK directly phosphorylates DAF-16 to promote its nuclear translocation and regulate its lifespan [25]. GLH-binding kinase 1 (KGB-1), which is encoded by *kgb-1* in *C. elegans*, is homologous to JNK. KGB-1 plays an important role in protecting *C. elegans* from internal and external stresses in an age-dependent manner. KGB-1 can protect *C. elegans* larvae from stress, but decreases stress resistance and shortens the lifespan in adults. DAF-16 is involved in the age-dependent regulation of *C. elegans* lifespan via KGB-1 [54,55]. In this study, the expression of *kgb-1* was significantly downregulated in *C. elegans* simultaneously treated with H_2_O_2_ and Hst, suggesting that Hst may inhibit *kgb-1* expression in adults to improve stress resistance and prolong lifespan.

In addition, previous studies have found that inhibiting the expression of *let-363* in neurons can prolong the lifespan of *C. elegans* [56]. The MAPK pathway is a typical longevity-regulating pathway [27], and DAF-12 can prolong the lifespan of *C. elegans*; additionally, coumarin enhances mitochondrial function and prolongs the lifespan of *C. elegans* by targeting DAF-12 [57], while nomilin significantly extends the lifespan and healthy lifespan of *C. elegans* and enhances toxin resistance through DAF-12 [58]. However, in *C. elegans*, *let-363* expression was significantly upregulated, while that of *pmk-2*, which belongs to the MAPK pathway, was significantly downregulated, and *daf-12* expression was significantly downregulated by Hst. Therefore, further studies are required to determine how the effects of Hst on the mTOR, MAPK, and DAF-12 pathways and chronic oxidative stress in *C. elegans* correlate with lifespan.

Our study has preliminarily demonstrated that Hst possesses the effect of anti-aging and anti-oxidative stress. Hst is the aglycone form of hesperidin and can be directly absorbed by enterocytes [59], so shows a higher absorbative ability than hesperidin. In addition, pharmacokinetic data have shown that Hst can be rapidly absorbed after oral administration, indicating better bioavailability than hesperidin. However, Hst still has the disadvantage of low bioavailability. Thus, how to apply Hst in the prevention and treatment of aging is a problem worthy of consideration. Its low bioavailability is partly due to the enzyme lysis of intestinal flora, resulting in the production of enzymatic hydrolysis products such as phenolic acid [60]. Therefore, the bioavailability of Hst can be improved by controlling intestinal flora in the future. Hst is also recognized as an inhibitor of cytochrome P450 [61], can increase the absorption of felodipine in the intestine, competitively inhibit felodipine metabolism in the liver, significantly change the pharmacokinetics of felodipine, and improve the therapeutic effects of felodipine for hypertension and angina [62], suggesting that Hst can be used in combination with other drugs to produce other pharmacological functions, thus improving the bioavailability of Hst. However, further research is required on the application and transformation of Hst.

## 4. Materials and Methods

### 4.1. Materials

Hst (97%), yeast extract powder, and glycerin were purchased from Shanghai Aladdin Biochemical Technology Co., Ltd. (Shanghai, China). Sodium chloride, agar powder, peptone, tryptone, DMSO, and enzyme-free aseptic water were purchased from Beijing Solaibao Biotechnology Co., Ltd. (Beijing, China). H_2_O_2_ and NaOH were purchased from Tianjin Jiangtian Chemical Technology Co., Ltd. (Tianjin, China). A sodium hypochlorite solution was obtained from Shanghai Maclin Biochemical Technology Co., Ltd. (Shanghai, China). The BCA Protein Assay Kit, ROS Assay Kit, Total Superoxide Dismutase Assay Kit with WST-8, and the Catalase Assay Kit were purchased from Shanghai Biyuntian Biotechnology Co., Ltd. (Shanghai, China). RNase-free EP tubes were purchased from Tianjin Dingguo Biotechnology Co., Ltd. (Tianjin, China). Liquid nitrogen was purchased from Tianjin Jinxi Huanda chemical gas Co., Ltd. (Tianjin, China).

### 4.2. C. elegans Strains and Maintenance

Wild-type *C. elegans* Bristol N2 was provided by the Caenorhabditis Genetics Center (St. Paul, MN, USA). *Escherichia coli* OP50 was a gift from Professor Wei-bin Ruan (College of Life Sciences, Nankai University, Tianjin, China). *C. elegans* was generally cultured at 20 °C on a solid nematode growth medium inoculated with OP50.

### 4.3. Synchronization of C. elegans

The medium contained a large number of oviposition nematodes, and the eggs were first washed with M9 buffer, centrifuged at 1000 rcf for 2 min, and this was repeated three times. The precipitate was dissolved with 1 mL of lysis buffer (M9 buffer: sodium hypochlorite: 5 M NaOH = 4:1:1), mixed and kept for 5 min, then centrifuged at 2500 rcf for 1 min; then dissolved with 1 mL M9 buffer and centrifuged at 2500 rcf for 3 min. This was repeated 3–4 times and the obtained eggs were transferred to NGM and cultured at 20 °C for 48 h to obtain L4 stage larvae.

### 4.4. Observation of the Influence of Hst and H_2_O_2_ on C. elegans Lifespan

First, we examined the effect of Hst on the lifespan of *C. elegans*. A total of 120 nematodes at the synchronous L4 stage were respectively transferred to solid NGM containing 0 μM, 50 μM, 75 μM, 100 μM, 150 μM, and 200 μM Hst and transferred to a new NGM containing different drug concentrations every other day. The numbers of live and dead nematodes were counted daily. When the nematode body was repeatedly touched with the platinum wire and showed no reaction, it was considered dead. The survival curve of the nematodes was plotted based on the number of deaths and days survived by the nematodes.

We examined the effects of H_2_O_2_ on the lifespan of *C. elegans*. A total of 120 nematodes at the synchronous L4 stage were transferred to solid NGM containing 0 μM, 200 μM, 400 μM, 800 μM, or 1 mM H_2_O_2_, and their lifespans were measured in the same way as described above.

All lifespan tests were independently repeated three times.

### 4.5. Determination of the Influence of Hst on the Frequency of Body Bending and Pharyngeal Pumping in H_2_O_2_-Treated C. elegans

Nematodes at synchronous stage L4 were transferred to NGM simultaneously containing Hst and H_2_O_2_ or only H_2_O_2_ for 3 and 8 d, respectively, and then transferred to NGM containing Hst or 0.1% DMSO for 5 and 10 d, respectively. The frequency of body bending or pharyngeal pumping of the nematodes over 30 s was observed and recorded under a microscope as a criterion for the locomotion or swallowing ability of the nematodes. At least 12 nematodes were observed and recorded for each treatment, and the experiment was independently repeated three times.

### 4.6. Determination of the Influence of Hst on the Antioxidant Ability of H_2_O_2_-Treated C. elegans

More than 1000 nematodes at stage L4 were transferred to solid NGM, simultaneously containing Hst and H_2_O_2_ or only H_2_O_2_. The method of measuring the anti-chronic oxidative stress ability was similar to that of the lifespan tests. All anti-chronic oxidative stress tests were independently repeated three times.

More than 1000 nematodes at synchronous stage L4 were respectively transferred to NGM simultaneously containing with Hst and H_2_O_2_ or only with H_2_O_2_ to culture for 3 and 5 d, then also transferred to NGM containing Hst or 0.1% DMSO for 3 and 5 d. ROS in different treated nematodes was determined according to the ROS Assay Kit, in which the following parameters were set: 37 °C, excitation wavelength of 488 nm, emission wavelength of 525 nm. The fluorescence values of the plates were measured every 20 min for 120 min. The SOD and CAT activities were also determined based on the absorbance values at 450 nm and 520 nm, respectively, in accordance with the manufacturer’s instructions. Each treatment group was set up with three holes, and the experiments were independently repeated three times.

### 4.7. Determination of the Influence of Hst on the Transcriptomic Metrics of H_2_O_2_-Treated C. elegans

More than 1800 nematodes at synchronous stage L4 were transferred to NGM simultaneously containing Hst and H_2_O_2_ or only H_2_O_2_ for 3 d, collected in 1.5 mL RNase-free EP tubes with M9 buffer, and centrifuged at 2500 rcf for 3 min, which was repeated three times. The samples of nematodes were immediately frozen by liquid nitrogen for more than 15 min, and then stored at −80 °C. The samples were sent to Beijing Qinglian Baiao Co., Ltd. (Beijing, China) for transcriptomic sequencing.

We removed low-quality areas and adapter sequences of the raw data from high-throughput sequencing to obtain high-quality data. The clean data were compared to the specified reference genome (WBcel235) using HISAT2 to obtain the efficiency of comparison between the sequencing data and the reference genome, the saturation of the sequencing data, and the gene coverage. Based on the results of the comparison, StringTie was used for transcript assembly, the gene expression levels in different samples were calculated, and gene expression profiles were constructed. DESeq2 was used to screen for differentially expressed transcripts in different sample groups, and Gene Ontology (GO) functional annotation and Kyoto Encyclopedia of Genes and Genomes (KEGG) enrichment analyses were performed for the differentially expressed transcripts.

### 4.8. Data Treatment and Statistical Analyses

GraphPad Prism version 8 for Windows (San Diego, CA, USA) was used for data processing and statistical analyses. The experimental results were presented as the mean ± standard error of the mean (SEM). The log-rank test was used to determine significant differences in lifespan and stress tests, and the Student’s *t*-test was used to analyze significant differences in other experiments. The cut-offs for statistical significance and extreme significance were set at *p* < 0.05 and *p* < 0.01, respectively.

## 5. Conclusions

Hst can increase the lifespan and antioxidative ability of *C. elegans* under chronic oxidative stress. Additionally, it can regulate the IIS, HSP, mtUPR, and JNK pathways, indicating that the increases in lifespan and antioxidant ability mediated by Hst are correlated with the IIS, HSP, mtUPR, and JNK pathways. However, quantitative reverse-transcription polymerase chain reaction and RNA interference tests are required to further verify the role of each key gene and determine whether Hst increases the lifespan and antioxidative ability during chronic oxidative stress in *C. elegans* via these signaling pathways.

## Figures and Tables

**Table 1:** The number of differentially expressed transcripts that resulted from different treatments.

Comparison Between Two Treatments	DETs	DETs (*p*adj ≤ 0.05, |Fold Change| ≥ 2)	UP	DOWN
control vs. H_2_O_2_	56,494	574	273	301
control vs. H_2_O_2_ + Hst	57,503	3590	2545	1045
H_2_O_2_ vs. H_2_O_2_ + Hst	56,901	1786	1265	521

Note: UP/DOWN is the number of differentially expressed transcripts upregulated or downregulated between two treatments.

**Table 2:** Some differentially expressed transcripts in chronic oxidative stress *C. elegans* treated with Hst.

Name		Description
*ist-1*		Homolog of insulin receptor substrate
*daf-16*		Homolog of forkhead box protein O
*daf-12*		Nuclear hormone receptor family
*daf-18*		Phosphatidylinositol 3,4,5-trisphosphate 3-phosphatase and dual-specificity protein phosphatase
*gst*	*gst-4*	Glutathione S-transferase
	*gst-3*	
	*gst-2*	
	*gst-39*	
*hsp*	*hsp-16.11*	Heat shock protein
*sip-1*		Stress induced protein
*let-363*		Homolog of rapamycin kinase mechanism target (m-TOR)
*clpp-1*		Homolog of caseinolytic mitochondrial matrix peptidase proteolytic subunit
*dve-1*		The transcription factor defective proventriculus
*kgb-1*		Homolog of Jun N-terminal kinase
*pmk-2*		Homolog of p38 mitogen-activated protein kinase

**Table 3:** The expression of some differentially expressed transcripts in chronic oxidative stress *C. elegans* treated with Hst.

Comparison Between Two Treatments	Gene ID	Gene Name	Transcript ID	log_2_FoldChange	*p*adj	Regulation
Control vs. H_2_O_2_ + Hst	WBGene00002163	*ist-1*	NewGene.26205.6	−26.04536394	2.74 × 10^−15^	down
	WBGene00000912	*daf-16*	R13H8.1a.1	12.73302285	3.22 × 10^−10^	up
			NewGene.2604.10	1.298891745	3.69 × 10^−5^	
	WBGene00000913	*daf-18*	T07A9.6.1	1.118064571	0.011809514	up
	WBGene00000908	*daf-12*	F11A1.3e.1	−2.754729785	0.000683622	down
	WBGene00001752	*gst-4*	K08F4.7.1	1.684174112	3.63 × 10^−6^	up
	WBGene00001787	*gst-39*	NewGene.7455.2	4.90335643	0.001169062	up
	WBGene00001751	*gst-3*	NewGene.14503.2	7.745929031	0.004127036	up
	WBGene00001750	*gst-2*	K08F4.6.1	6.754003888	0.011318526	up
	WBGene00002017	*hsp-16.11*	NewGene.21283.1	30	1.59 × 10^−19^	up
	WBGene00004798	*sip-1*	F43D9.4.1	1.203440588	0.002276358	up
	WBGene00002583	*let-363*	NewGene.1055.6	1.290698592	0.022614399	up
	WBGene00014172	*clpp-1*	ZK970.2a.1	4.700864293	0.005367563	up
	WBGene00022861	*dve-1*	ZK1193.5d.4	3.278749313	0.021807511	up
	WBGene00002187	*kgb-1*	T07A9.3.2	−2.182066809	0.020845379	down
			T07A9.3.3	−2.758890155	0.023211212	
H_2_O_2_ vs. H_2_O_2_ + Hst	WBGene00002163	*ist-1*	NewGene.26205.6	−10.83449562	0.000992388	down
	WBGene00000912	*daf-16*	NewGene.2604.10	1.501111364	9.97 × 10^−6^	up
	WBGene00001787	*gst-39*	NewGene.7455.2	6.0306605	0.001272731	up
	WBGene00001750	*gst-2*	K08F4.6.1	6.658125532	0.026258173	up
	WBGene00002017	*hsp-16.11*	NewGene.21283.1	30	7.41 × 10^−20^	up
	WBGene00002583	*let-363*	NewGene.1055.6	1.384233821	0.040471686	up
	WBGene00014172	*clpp-1*	ZK970.2a.1	5.269072011	0.002155423	up
	WBGene00004056	*pmk-2*	F42G8.3c.1	−2.695909789	0.022225121	down
	WBGene00002187	*kgb-1*	T07A9.3.3	−3.558758022	0.001013018	down
			T07A9.3.2	−2.564149218	0.006242272	

## Data Availability

The original contributions presented in this study are included in the article. Further inquiries can be directed to the corresponding authors.
